# Modified Timed Up and Go Test for Tendency to Fall and Balance Assessment in Elderly Patients With Gait Instability

**DOI:** 10.3389/fneur.2020.00543

**Published:** 2020-06-12

**Authors:** Andrés Soto-Varela, Marcos Rossi-Izquierdo, María del-Río-Valeiras, Ana Faraldo-García, Isabel Vaamonde-Sánchez-Andrade, Antonio Lirola-Delgado, Sofía Santos-Pérez

**Affiliations:** ^1^Division of Neurotology, Department of Otorhinolaryngology, Complexo Hospitalario Universitario, Santiago de Compostela, Spain; ^2^Department of Surgery and Medical-Surgical Specialities, University of Santiago de Compostela, Santiago de Compostela, Spain; ^3^Department of Otorhinolaryngology, University Hospital Lucus Augusti, Lugo, Spain; ^4^Department of Otorhinolaryngology, Complexo Hospitalario Universitario, Santiago de Compostela, Spain

**Keywords:** Timed Up and Go, falls in elderly, computerized dynamic posturography, mobile posturography, Dizziness Handicap Inventory, short FES-I

## Abstract

**Objective:** To compare the results from the modified Timed Up and Go Test (TUG) with posturographic variables, the subjective perception of disability due to gait instability, and the number of falls in a sample of the elderly population with imbalance, to confirm that the TUG Test is a useful clinical instrument to assess the tendency to fall in individuals of this age group.

**Materials and Methods:** Cross-sectional study conducted in a tertiary university hospital, in 174 people aged 65 years or older with gait instability. Modified TUG Test was performed; time, step count and the need for support during the test were the analyzed variables. They were compared with the number of falls, Computerized Dynamic Posturography scores, and questionnaires scores (Dizziness Handicap Inventory and a shortened version of the Falls Efficacy Scale-International).

**Results:** The average time to complete the TUG Test was 21.24 ± 8.18 s, and the average step count was 27.36 ± 7.93. One hundred two patients (58.6%) required no support to complete the test, whereas the other 72 (41.4%) used supports. The time taken to complete the Test was significantly related with having or not having fallen in the previous year, with the scores of the questionnaires, and with various parameters of dynamic posturography. A higher percentage of patients who took more than 15 s had fallen in the previous year than those who took up to 15 s to complete the test [*P* = 0.012; *OR* = 2.378; 95% CI (1.183, 4.780)]. No significant correlation was found between the step count and the number of falls in the previous year, with falling during the test or not, or with being a single or a frequent faller. No relation was found between the need for supports and the number of falls, with having or not having fallen in the previous year, or with being a single or frequent faller.

**Conclusion:** The modified TUG Test is in relation with the presence or absence of falls. Time is the essential parameter for analyzing the risk of falling and the 15-s threshold is a good value to differentiate elderly patients at high risk of falling.

**Unique Identifier:** NCT03034655, www.clinicaltrials.gov.

## Introduction

Balance disorders are one of the factors that prompt falls in the elderly and are a major socio-sanitary problem in terms of morbidity and mortality and over expenditure by healthcare systems and by the families themselves (1, 2). The causes of balance disorders in the elderly are numerous (neurological, vestibular system and locomotor disorders, loss of strength and flexibility, the aging of the sensory systems that contribute to maintaining stability, etc.) (3, 4).

Although this is an aspect on which there is still no definitive consensus, it is considered that exercise interventions appear to be effective using a wider range of types of exercise than are currently recommended (5). The optimum approach for older people living in the community with risk of falls should include strength and balance exercises (4, 6, 7). Balance can be improved in the elderly by using vestibular rehabilitation protocols (8–10). These protocols also contribute to increased scores on balance tests (such as computerized dynamic posturography), and moreover, to a decrease in the number of falls (11, 12). However, subjecting all of the elderly to a vestibular rehabilitation protocol is unfeasible. Therefore, instruments must be developed to identify those at high risk for falls, thereby selectively helping them improve their stability. Assessing balance using tools such as dynamic posturography has efficiently identified elderly people at risk for falls (13). Although performing posturography on all the elderly is also not possible, applying a clinical examination instrument for an initial selection of individuals at high risk of falls would be useful.

The Timed Up and Go (TUG) Test (14) is a clinical test in which gait is assessed without using any instrument. Only one chair and a surface for the patient to walk three meters are required. The duration of the test, including an explanation regarding the patient's performance, is <1 min. Although some authors have suggested that a long test performance time is an indicator of a high risk for falls (15), this association has not been clearly demonstrated (16).

The objective of the present study was to compare the results from the TUG Test with posturographic variables, the subjective perception of disability due to gait instability, and the number of falls in a sample of the elderly population with imbalance, to confirm that the TUG Test is a useful clinical instrument to assess the tendency to fall in individuals of this age group.

## Materials and Methods

This study is part of a larger research project, which aims to evaluate the usefulness of vestibular rehabilitation in elderly people with instability, to improve balance and reduce falls. The complete protocol of this research project has been published (17).

### Study Design

This cross-sectional study was conducted in a tertiary university hospital.

#### Study Population Inclusion and Exclusion Criteria

Patients older than 65 years with gait instability, who met at least two of the following inclusion criteria were included in the study: (a) fell at least once in the previous 12 months, (b) used more than 15 s or needed support during the TUG Test, (c) obtained a mean computerized dynamic posturography (CDP) sensory organization test (SOT) balance score <68%, (d) fell at least once in the CDP SOT, or (e) had a score >60% in Vertiguard's geriatric Standard Balance Deficit Test. The following exclusion criteria were considered: (a) cognitive decline or reduced cultural level that prevents the patient from understanding the assessment or granting informed consent, (b) organic conditions that prevent standing on two feet, necessary for an assessment of balance and performance of vestibular rehabilitation exercises, (c) balance disorders caused by conditions other than age (neurologic, vestibular, etc.), or (d) current treatment with drugs which may alter balance.

#### Sample

In total, 174 people aged 65 years or older met the inclusion criteria, had visited the Otoneurology Unit of a tertiary hospital for balance alterations, and were included in the study. The average age was 77.40 ± 6.35 years, with a maximum age of 92. Regarding the distribution by sex, the sample was divided into 132 women (75.9%) and 42 men (24.1%); the female/male ratio was 3.1/1.

As regards the inclusion criteria, 115 patients met the criterion (a) (fell at least once in the previous 12 months), 139 met the criterion (b) (used more than 15 s or needed support during the TUG Test), 150 met the criterion (c) (obtained a mean CDP SOT balance score <68%), 153 met the criterion (d) (fell at least once in the CDP SOT) and 24 met the criterion (e) (had a score >60% in Vertiguard's geriatric Standard Balance Deficit Test). Thirty-nine patients were included with two inclusion criteria, 46 with three, 78 with four, and 11 with five.

### Method

To exclude a pathological cause of impaired balance, all subjects underwent a complete clinical otoneurological assessment, which included a neurological examination. Specifically, the head impulse test (if saccades of refixation were present, the patient was excluded of the study) and the exploration of the nystagmus using Frenzel glasses (whether spontaneous or through the head shaking or the Dix-Hallpike test) were performed. When necessary, videonystagmography and caloric tests, video head impulse tests, vestibular evoked potentials, and/or brain magnetic resonance imaging were also recorded.

To evaluate their balance and to assess whether they met the inclusion criteria, the following examinations and balance tests were performed:

a) A modified TUG Test (18, 19): the subject, seated, had to stand up without aid, walk 3 meters, turn around and sit down again. The time needed, numbers of steps taken, and any use of support were recorded.
b) The computerized dynamic posturography (CDP) sensory organization test (SOT); we used the Neurocom® Smart Equitest platform. The SOT included quantitation of the patient's center of pressure (COP) displacements in six different sensorial information conditions:
  1: fixed surface and visual surround, eyes open
  2: fixed surface, eyes closed
  3: fixed surface, eyes open, moving visual surround
  4: moving surface, eyes open, fixed visual surround
  5: moving surface, eyes closed
  6: moving surface, eyes open, moving visual surround.
  Each of the six conditions was repeated three consecutive times, with the patients completing a total of 18 tests per entry. The time established for each of these tests was 20 s.
c) CDP limits of stability (LOS). Following visual feedback (movement of a pictogram representing the subject's COP on a TV monitor), the patient had to voluntarily move his or her COP without moving his or her feet on the platform, to reach eight points around him/her. These points represented 100% of the displacement limit of the subject's COP, according to height and age.
d) The mobile Vertiguard® system: 14 tests were performed and their analyses represented the geriatric Standard Balance Deficit Test:
  - Standing still (SS), eyes open, normal surface (NS)
  - SS, eyes closed, NS
  - SS, one leg, eyes open, NS
  - 8 steps in tandem, eyes open, NS
  - SS, eyes open, foam surface (FS)
  - SS, eyes closed, FS
  - 8 steps in tandem, eyes open, FS
  - Walk 3 m, eyes open
  - Walk 3 m, eyes open, turning the head from side to side
  - Walk 3 m, eyes open, moving the head up and down
  - Walk 3 m, eyes closed
  - Walk over 4 barriers (height: 26 cm; distance between barriers: 1 m)
  - Sit down on a chair
  - Get up from a chair.
e) Questionnaires that measured disability resulting from imbalance and the risk of falling:
  - Direct question about the number of falls in the previous 12 months
  - Dizziness Handicap Inventory (DHI), validated in Spanish (20): assessed disability perceived by the patient in relation to instability. It consisted of 25 items divided into 3 groups (9 on the functional scale, 9 on the emotional scale, and 7 on the physical scale), with 3 possible answers: “yes” (4 points), “sometimes” (2 points), and “no” (0 points). The highest perception of disability would be 100 and the lowest would be 0.
  - A shortened version of the Falls Efficacy Scale-International (Short FES-I) to assess the fear of falling (21): it evaluated the fear of falling while performing seven everyday activities. There were four possible answers: “not at all concerned” (0 points), “somewhat concerned” (1 point), “quite concerned” (2 points), and “very concerned” (3 points). The highest score (greatest fear of falling) would be 21 and the lowest would be 0.

The balance tests (TUG, CDP, and Vertiguard) were carried out by trained personnel in the vestibular exploration. In all of them, the patient is previously explained what each test consists of and a first training record is made. Next, the tests were carried out according to the protocol followed in our clinic: three tests in each task for CDP SOT, and one for TUG, CDP LOS, and gSBDT Vertiguard. The questionnaires were delivered in writing to the patient (after an explanation by the researcher), who answers them on their own or with the help of a family member.

#### Study Variables

The following variables were recognized and analyzed:

a) Age and sex
b) TUG Test:
  - Time: was analyzed as a continuous variable and as a discrete variable. For such purposes, two different cutoff points were established:
  - Up to 15 s and more than 15 s (time established as one of the inclusion criteria).
  - Below and above the average time spent (which, as indicated in the Results section, was 21.2 s).
  - Step count: was analyzed as a continuous variable and as a discrete variable. The discrete variable was determined by dividing the patients into two groups, and the average number of precise steps required to complete the test (which was 27.4) was defined as the cutoff point.
  - The need for supports (or not).
c) SOT by dynamic posturography:
  - The percentage score for each condition (the arithmetic mean of the three entries for each condition).
  - The overall average balance, which was calculated as the weighted average of the 18 SOT scores.
  - The number of falls that occurred when completing the 18 SOTs.
  - The effectiveness of somatosensory information use, which was a percentage value from the application of the following formula: (average score of condition 2/average score of condition 1) × 100.
  - The effectiveness of visual input use, which was calculated using the following formula: (average score of condition 4/average score of condition 1) × 100.
  - The effectiveness of vestibular input use, which was assessed using the following calculation: (average score of condition 5/average score of condition 1) × 100.
  - The ability to assume erroneous visual input, a score was assigned using the following calculation made using the values determined by the conditions: [(2 + 5)/(3 + 6)] × 100.
d) CDP LOS:
  - Reaction time: time from onset of visual signal showing movement to its actual beginning (in seconds).
  - Movement velocity: mean speed of displacement from COP, as °/s.
  - Maximum excursion (ME): measurement of the maximum COP excursion, with respect to 100% of the theoretical limit of stability (as a percentage).
  - Endpoint excursion (EE): measure of the point reached at the end of the displacement of the COP, relative to the theoretical 100% of limit of stability (as a percentage).
  - Directional control: comparison between movement in the direction of the target vs. movement away from that direction, as a percentage.
e) Questionnaires:
  - The number of falls in the last year, which was analyzed as a continuous variable and as a discrete variable. For such purposes, the patients were divided into fallers (at least one fall in the previous year) vs. non-fallers (no fall), and into single-fallers (up to five falls) vs. frequent fallers (more than five falls).
  - DHI score, total score, and the score of each scale (emotional, functional, and physical)
  - Short FES-I score.

#### Statistical Analysis

The Kolmogorov-Smirnoff test was used to assess whether different quantitative variables followed a normal distribution. To assess association between qualitative variables (sex, fallers vs. non-fallers, single-fallers vs. multiple fallers, need for support in TUG test, more or <15 s in TUG test, more or <21.2 s in TUG test, and more or <27.4 steps in TUG test), always in 2 × 2 tables, Fisher's exact test was used, showing the odds ratio (OR) and 95% confidence intervals (CI). To compare quantitative (age, time and steps in TUG tests, CDP scores, and questionnaires scores) and qualitative variables, when the former followed a normal distribution, Student's *t* was used; when the distribution was not normal, the non-parametric Mann-Whitney test was used. Finally, to correlate quantitative variables with each other, Spearman's Rho correlation test was used. The level of statistical significance in all tests was set at *P* < 0.05.

The software SPSS 15.0 for Windows was used for the statistical analysis.

### Ethical Aspects

The protocol has been approved by the Independent Ethics Committee of Galicia (protocol 2014/411).

The study was conducted according to ICH Good Clinical Practices and the Declaration of Helsinki and Law 14/2007 on July 3, on Biomedical Research. All patients gave their written consent to participate in the study.

## Results

As indicated above, 174 patients, were included in the study. No significant differences in age were found between the sexes (with a mean age of 77.25 years in women and of 77.88 years in men; *P* = 0.576, Student's *t*-test).

For the total sample, the average time that the patients took to complete the TUG Test was 21.24 ± 8.18 s, and the average step count was 27.36 ± 7.93. In this study, 102 patients (58.6%) required no supports to complete the test, whereas the other 72 (41.4%) needed supports. Significant differences in the average time were found between the sexes (22.09 s in women vs. 18.54 in men; *P* = 0.009, Mann-Whitney test) and in the average step count (28.23 in women vs. 24.62 in men; *P* = 0.003, Mann-Whitney test), but not in the need for supports (*P* = 0.481, Fisher's exact test).

The time taken to complete the TUG Test was significantly related with having or not having fallen in the previous year, with the scores of the questionnaires, and with various parameters of dynamic posturography; all these associations are shown in Table 1. Although approaching statistical significance, no correlation was found in relation to the total number of falls in the previous year (*P* = 0.051, coefficient value = 0.148, Spearman's Rho correlation), or with being a single or a frequent faller (*P* = 0.896, Mann-Whitney test).

**Table 1:** Variables significantly related to the time taken to complete the Timed Up and Go Test.

**Parameter**			***p*-value (coefficient value)**	**Statistical test**
Falls in the previous year	Falls vs. non-falls			
	Falls (average ± SD)	Non-falls (average ± SD)	0.021	Mann-Whitney
	22.11 ± 9.02 s	14.53 ± 5.91 s		
CDP sensory organization test	Overall average balance		0.013 (−0.189)	Spearman's Rho
	Condition 5		0.037 (−0.158)	Spearman's Rho
	Condition 6		0.005 (−0.214)	Spearman's Rho
	Vestibular input		0.044 (−0.153)	Spearman's Rho
	Number of falls		<0.001 (0.264)	Spearman's Rho
CDP limits of stability	Movement velocity		0.026 (−0.169)	Spearman's Rho
	Endpoint excursion		<0.001 (−0.265)	Spearman's Rho
	Maximum excursion		<0.001 (−0.328)	Spearman's Rho
	Directional control		<0.001 (−0.346)	Spearman's Rho
DHI	Total score		<0.001 (0.323)	Spearman's Rho
	Physical scale		0.002 (0.238)	Spearman's Rho
	Emotional scale		0.003 (0.220)	Spearman's Rho
	Functional scale		<0.001 (0.349)	Spearman's Rho
Short FES-I	Score		<0.001 (0.502)	Spearman's Rho

When dividing the patients into two groups based on time (up to 15 s vs. more than 15 s), the results showed a significant association with having or not having fallen in the previous year (Table 2). Specifically, a higher percentage of those who took more than 15 s had fallen in the previous year than those who took up to 15 s to complete the test [*P* = 0.012; *OR* = 2.378; 95% CI (1.183, 4.780); Fisher's exact test]. The results also show a relation with the number of falls (*P* = 0.009, Mann Whitney test), but not with being a single or a frequent faller (*P* = 0.115, Fisher's exact test). The patients who took up to 15 s to complete the test had higher scores in various posturographic parameters and in the questionnaires, as shown in Table 3.

**Table 2:** Relationship between the time taken to complete the Timed Up and Go Test and the number of patients having fallen or not in the previous year.

	**No falls**	**Falls**	**Total**
Up to 15 s	22	23	45
More than 15 s	37	92	129
Total	59	115	174

**Table 3:** Variables significantly related to taking more or <15 s to complete the Timed Up and Go Test.

**Parameter**		**>15 s (average ± SD)**	** <15 s (average ± SD)**	***p*-value**	**Statistical test**
Falls in the previous year	Falls vs. no falls			0.012	Fisher's exact test
	Number of falls^*^	10.98 ± 54.65	5.22 ± 12.09	0.009	Mann-Whitney
CDP sensory organization test	Condition 6^**^	21.19 ± 21.98	29.27 ± 22.20	0.020	Mann-Whitney
	Number of falls^*^	3.63 ± 2.54	2.58 ± 2.23	0.017	Mann-Whitney
CDP limits of stability	Movement velocity^**^	2.29 ± 0.80	2.46 ± 0.63	0.032	Mann-Whitney
	Endpoint excursion^**^	47.09 ± 10.94	55.91 ± 13.37	<0.001	Student's *t*-test
	Maximum excursion^**^	64.02 ± 11.90	73.78 ± 12.38	<0.001	Mann-Whitney
	Directional control^**^	63.02 ± 12.51	73.24 ± 9.67	<0.001	Student's *t*-test
DHI	Total score^*^	59.09 ± 19.82	43.64 ± 23.53	<0.001	Mann-Whitney
	Physical scale^*^	17.92 ± 6.58	13.82 ± 7.99	0.004	Mann-Whitney
	Emotional scale^*^	16.62 ± 8.92	12.80 ± 9.39	0.016	Student's *t*-test
	Functional scale^*^	24.54 ± 8.44	17.02 ± 9.97	<0.001	Mann-Whitney
Short FES-I	Score^*^	10.48 ± 4.96	5.73 ± 4.34	<0.001	Mann-Whitney

* Higher scores, related to more than 15 s;

** *higher scores, related to <15 s; falls, related to more than 15 s*.

When dividing the two groups by the cutoff point of 21.2 s for the average time of the sample, needing less than this time to complete the test was significantly related with not having fallen in the previous year [*P* = 0.012; *OR* = 2.239; 95% CI (1.152, 4.348); Fisher's exact test] and with the variables included in Table 4.

**Table 4:** Variables significantly related to taking more or <21.2 s to complete the Timed Up and Go Test.

**Parameter**		**>21.2 s (average ± SD)**	** <21.2 s (average ± SD)**	***p*-value**	**Statistical test**
Falls in the previous year				0.012	Fisher's exact test
CDP sensory organization test	Overall average balance^**^	51.87 ± 11.80	56.64 ± 12.37	0.011	Student's *t*-test
	Condition 5^**^	21.89 ± 22.25	28.72 ± 23.39	0.045	Mann-Whitney
	Condition 6^**^	18.70 ± 20.31	26.75 ± 23.12	0.015	Mann-Whitney
	Input vestibular^**^	24 ± 24.33	31.15 ± 25.22	0.049	Mann-Whitney
	Number of falls^*^	4.08 ± 2.56	2.81 ± 2.33	0.001	Mann-Whitney
CDP limits of stability	Endpoint excursion^**^	46.53 ± 11.06	51.53 ± 12.64	0.006	Student's *t*-test
	Maximum excursion^**^	62.81 ± 12.69	39.37 ± 12.07	0.001	Student's *t*-test
	Directional control^**^	62.12 ± 12.93	68.34 ± 11.78	0.001	Student's *t*-test
DHI	Total score^*^	61.17 ± 18.61	50.48 ± 23.06	0.002	Mann-Whitney
	Physical scale^*^	18.67 ± 6.38	15.49 ± 7.47	0.006	Mann-Whitney
	Emotional scale^*^	17.12 ± 8.62	14.51 ± 9.45	0.043	Mann-Whitney
	Functional scale^*^	25.39 ± 7.90	20.48 ± 9.97	0.002	Mann-Whitney
Short FES-I	Score^*^	11.73 ± 4.55	7.37 ± 4.93	<0.001	Mann-Whitney

* Higher scores, related to more than 21.2 s;

** *higher scores, related to <21.2 s; falls, related to more than 21.2 s*.

When analyzing the step count needed to complete the TUG Test, no significant correlation was found with the number of falls in the previous year (*P* = 0.095, coefficient value = 0.127, Spearman's Rho), with falling during the test or not (*P* = 0.097, Mann-Whitney test), or with being a single or a frequent faller (*P* = 0.629, Mann-Whitney test). However, the step count was significantly correlated with different variables (posturographic parameters and questionnaires), which are outlined in Table 5.

**Table 5:** Variables significantly related to the step count in completing the Timed Up and Go Test.

**Parameter**		***p*-value (coefficient value)**	**Statistical test**
CDP sensory organization test	Overall average balance	0.002 (−0.232)	Spearman's Rho
	Condition 5	0.007 (−0.204)	Spearman's Rho
	Condition 6	<0.001 (−0.302)	Spearman's Rho
	Vestibular input	0.008 (−0.201)	Spearman's Rho
	Number of falls	<0.001 (0.343)	Spearman's Rho
CDP limits of stability	Endpoint excursion	0.001 (−0.259)	Spearman's Rho
	Maximum excursion	<0.001 (−0.295)	Spearman's Rho
	Directional control	<0.001 (−0.383)	Spearman's Rho
DHI	Total score	<0.001 (0.267)	Spearman's Rho
	Physical scale	0.031 (0.164)	Spearman's Rho
	Emotional scale	0.003 (0.225)	Spearman's Rho
	Functional scale	<0.001 (0.279)	Spearman's Rho
Short FES-I	Score	<0.001 (0.467)	Spearman's Rho

When dividing the patients into two groups based on the step count (more or <27.4 steps, the average value of the sample), no significant association was found with the number of falls in the previous year (*P* = 0.069, Mann-Whitney test), with having or not having fallen in the previous year (*P* = 0.067, Fisher's exact test), or with being a single or frequent faller (*P* = 0.415, Fisher's exact test). The relationships with other study variables are shown in Table 6.

**Table 6:** Variables significantly related to taking more or <27.4 steps to complete the Timed Up and Go Test.

**Parameter**		**>27.4 steps (average ± SD)**	** <27.4 steps (average ± SD)**	***p*-value**	**Statistical test**
CDP sensory organization test	Overall average balance^**^	51.39 ± 11.63	56.78 ± 12.36	0.004	Student's *t*-test
	Condition 5^**^	21.70 ± 22.40	28.58 ± 23.24	0.045	Mann-Whitney
	Condition 6^**^	16.46 ± 19.75	27.98 ± 22.75	<0.001	Mann-Whitney
	Number of falls^*^	4.24 ± 2.52	2.75 ± 2.32	<0.001	Mann-Whitney
CDP limits of stability	Endpoint excursion^**^	46.38 ± 10.51	51.44 ± 12.89	0.005	Student's *t*-test
	Maximum excursion^**^	62.83 ± 12.10	69.11 ± 12.58	0.001	Student's *t*-test
	Directional control^**^	61.39 ± 13.31	68.60 ± 11.31	0.001	Mann-Whitney
DHI	Total score^*^	60.25 ± 18.02	51.53 ± 23.57	0.006	Student's *t*-test
	Emotional scale^*^	17.44 ± 8.21	14.39 ± 9.62	0.015	Mann-Whitney
	Functional scale^*^	24.82 ± 7.98	21.07 ± 10.07	0.023	Mann-Whitney
Short FES-I	Score^*^	11.59 ± 4.81	7.64 ± 4.91	<0.001	Mann-Whitney

* Higher scores, related to more than 27.4 steps;

** *higher scores, related to <27.4 steps*.

Lastly, when analyzing the need for supports or not, no relation was found with the number of falls (*P* = 0.814, Mann-Whitney test), with having or not having fallen in the previous year (*P* = 0.488, Fisher's exact test), or with being a single or frequent faller (*P* = 0.079, Fisher's exact test). Significant associations of this variable with other posturographic and questionnaire variables were scarce: with endpoint and maximum excursion of LOS (*P* = 0.01 and P = 8.655 e^−5^, respectively; Student's *t*-test), and with the directional control (*P* = 0.002, Mann-Whitney test) of the LOS as well.

## Discussion

Balance disorders, common among the elderly, are a cause of falls in this age group (22). Detecting individuals at high risk for falls would allow us to help them through rehabilitation strategies to improve their balance and thus reduce the likelihood of falls. Posturography has proven to be useful in identifying these patients (13); however, performing posturography on all the elderly as a screening method for detecting the risk of falls is unrealistic from a clinical standpoint. A non-invasive and fast clinical test requiring no instruments would be ideal for pre-selecting patients at risk for falls in primary care. For this purpose, different clinical tests and questionnaires have been evaluated: a prior history of falls, the TUG test, the sit-to-stand test with one and five repetitions, the pick-up-weight test, the half-turn test, the alternate-step test, the six-meter-walk test, the stair ascent and descent tasks, the Berg Balance Scale, the 3-m zigzag walk test, the EuroQol EQ-5D questionnaire, and the short FES-I scale (23–28).

The TUG Test was described in 1991 by Podsiadlo and Richardson (14). A patient, initially sitting in a chair, gets up, walks three meters, turns around, goes back to the chair, and sits again. Taking 10 s or longer to complete the test was reported as an indicator of mobility impairment (14, 29). Subsequently, Vaillant et al. (18) introduced a modification to increase the difficulty of the test: upon returning to the chair, the patient had to go around it and sit on it. Logically, the time required to perform the test would then be longer. This latest version was used in our study. The TUG is a fast and simple way of assessing gait and has been considered a good indicator of the risk for falls (15). However, this correlation has not been clearly established in previous studies (13). In turn, high scores in the TUG have been related with the risk of dementia (30).

In this study, several parameters of the modified TUG (time, precise step count, and need for a support or not) have been correlated with having fallen in the previous 12 months (key parameter) and also with posturography and questionnaire variables.

First, the TUG parameter with the strongest relation with falls is the time. Neither the step count nor the need for supports showed any relationship with the falls suffered in the previous year. The irrelevance of the need for supports is especially striking because it is not associated with the subjective perception of gait instability either, as measured by the questionnaires, or exclusively with posturographic values (only with the endpoint and maximum excursion of the LOS). Perhaps patients who support themselves during the TUG Test are more careful when moving in their daily life, and therefore, fall less frequently. In any case, the need for supports in the TUG Test is apparently not related with the tendency to fall among elderly patients with gait instability.

The step count during the TUG Test is not related with having fallen in the previous year either. People who have fallen more frequently do not need more steps to complete the test, as a priori might seem likely. However, the step count is significantly related to many posturographic variables (with the overall average balance and with the use of vestibular input) and with all the parameters of the questionnaires. Specifically, the step count is very significantly related to the fear of falling (as assessed by the short FES-I score), and this relationship could explain precisely why they do not fall: subjects with a high perception of disability due to their gait instability (measured with the DHI) and with a fear of falling (measured with the FES-I short) have a higher step count when walking, thereby increasing safety and reducing the number of falls. It is striking the fact that the time required for the TUG-test but not the number of steps is indicative for identifying fallers. One possible reason for this finding could be a larger step length for the non-fallers.

The key result of our study was the confirmation that time is the parameter that correlates most robustly with falls. Although the time cutoffs set (15 and 21.2 s) differentiate patients who have fallen from those who have not, the division between those who take more or <15 s to complete the TUG Test is the most associated with the number of falls. In parallel, completing the TUG Test quickly is strongly correlated with the posturographic scores and questionnaire parameters (especially in the short FES-I), in line with previous studies, which have indicated that posturography scores (8) and the short FES-I score (25) are good predictors of the risk for falls (31).

The main limitation of this study was that the number of falls was quantified retrospectively. This involves a memory bias: patients can easily remember whether they have fallen or not in the last year, but if they have indeed fallen, they may not remember precisely how many times it happened. Since there was no relation found between any of the parameters and being a single or frequent faller, this is likely due to a lack of precision in the quantification of the falls.

Another limitation is the fact that one of the possible inclusion criteria is the time used in the TUG test. In fact, one of the aspects that we sought to answer in this study is to confirm if 15 seconds is effectively a good cut-off point. Not all patients included in this study take more than 15 s to perform the TUG test, because many of them meet other inclusion criteria. For all these reasons, we believe that including TUG time as one of the possible inclusion criteria should not be a bias, at least not relevant.

The essential conclusion was that the modified TUG Test is a clinical exploration instrument that correlates well with the presence or absence of falls. Time is the essential parameter for analyzing the risk of falling (step count and the need for a support or not are less relevant) and the 15-s threshold is a good value to differentiate elderly patients with high tendency to fall.

## Data Availability Statement

The datasets generated for this study are available on request to the corresponding author.

## Ethics Statement

The studies involving human participants were reviewed and approved by Independent Ethics Committee of Galicia (protocol 2014/411). The patients/participants provided their written informed consent to participate in this study.

## Author Contributions

AS-V, MR-I, MR-V, AF-G, IV-S-A, AL-D, and SS-P have contributed to the conception and design of this manuscript, revised it critically, approved the final version and agree to be accountable for all aspects of the work in ensuring that questions related to the accuracy or integrity of any part of the work are appropriately investigated and resolved. AS-V has designed the protocol of the study and written the manuscript. AF-G, IV-S-A, and AL-D have performed the clinical and posturographic examination. MR-V has collected and analyzed the data. MR-I has developed the statistical analysis. SS-P has revised critically the manuscript. All authors contributed to the article and approved the submitted version.

## Conflict of Interest

The authors declare that the research was conducted in the absence of any commercial or financial relationships that could be construed as a potential conflict of interest.
